# Urate oxidase from tea microbe *Colletotrichum camelliae* is involved in the caffeine metabolism pathway and plays a role in fungal virulence

**DOI:** 10.3389/fnut.2022.1038806

**Published:** 2023-01-04

**Authors:** Shengnan He, Xiaoyan Qiao, Shuhan Zhang, Jinglin Xia, Lei Wang, Shouan Liu

**Affiliations:** ^1^Laboratory of Tea and Medicinal Plant Biology, College of Plant Sciences, Jilin University, Changchun, China; ^2^Guangdong Provincial Key Laboratory of Tea Plant Resources Innovation and Utilization/Guangdong Academy of Agricultural Sciences Tea Research Institute, Guangzhou, China; ^3^Institute of Chemical and Industrial Bioengineering, Jilin Engineering Normal University, Changchun, China; ^4^Laboratory of Molecular Plant Pathology, College of Plant Sciences, Jilin University, Changchun, China

**Keywords:** *Colletotrichum camelliae*, tea plant, caffeine metabolism, urate oxidase, purine alkaloids

## Abstract

Tea is one of the most well-known, healthy beverages in the world. Tea plants produce caffeine as a secondary metabolite. *Colletotrichum camelliae* is one of the most important microbes frequently isolated from tea fields, and it causes anthracnose disease in tea plant. In the present work, we performed molecular microbiology and transcriptomic analyses of the *C. camelliae* - tea plant interaction to investigate the mechanism of fungal virulence and plant defense. Upon infection of tea plant with *C. camelliae*, we observed alterations in the expression of fungal transcripts, including those of many genes associated with caffeine metabolism, such as those encoding various transporters, xanthine dehydrogenase, and urate oxidase (UOX). In particular, the deletion of *C. camelliae* urate oxidase (*CcUOX*), which is involved in the caffeine metabolism pathway, reduced fungal tolerance to caffeine, and impaired fungal virulence. CcUOX is involved in caffeine metabolism by the degradation of uric acid contents. *C. camelliae*Δ*CcUOX* mutants impaired uric acid degradation *in vivo*. The *CcUOX* gene was cloned from *C. camelliae*, overexpressed in *Escherichia coli*, and the recombinant CcUOX protein displayed maximum activity at 30°C and a pH of 4.0. The recombinant CcUOX efficiently reduced uric acid *in vitro* suggesting a promising application in caffeine-contaminated environment management and in producing food with low purine contents to prevent uric acid related human diseases, such as hyperuricemia and gout.

## 1. Introduction

Tea, together with cocoa and coffee, are the three most well-known, healthy, non-alcoholic beverages worldwide. Tea plant *Camellia sinensis* (L.) O. Kuntze is derived from southwestern China and now tea industry provides plentiful wealth and job opportunities in more than 50 countries (1). Tea plants are perennial woody plants with an economic life span of 40–50 years. During their life cycle, tea plants face multiple environmental stresses, including pathogens, insects and abiotic stresses (1, 2). Among these pathogens, *Colletotrichum* spp. cause anthracnose, which usually occurs on tea leaves and ultimately influences tea yield and quality (3–6). Upon *Colletotrichum* infection, bottle-green, watery lesions emerged on the surface of tea leaves at an early stage, and the scabs enlarged over time (4). At the late phase, dense tiny black dots called acervuli appear on the lesion, which produce conidia that facilitates disease transmission and causes disease spread (4). *Colletotrichum camelliae* is an important fungal microbe in tea fields, and previous studies have proposed that it may have evolved alongside tea plants (3, 4). Recently, *C. camelliae* has been indicated to be one of the dominant fungal pathogens in tea plants of China owing to its high isolation rate in tea production regions and strong pathogenicity (3–6).

Tea plants contain abundant caffeine (1,3,7-trimethylxanthine), which has antimicrobial effects against various pathogens (4, 5, 7, 8). Plant resistance to pathogens may depend on the effects of caffeine, which is hypothesized to act as a pesticide (9). However, there are many ways by which caffeine enters the environment and thus exhibits negative effects on the surrounding environment (10–12). The releasing of caffeine into the soil and groundwater may arise from tea fallen leaves, stems, seeds, and also from the liquid and solid wastes in processed tea or coffee materials (10). In addition, the accumulation of caffeine in the natural environment may be the result of increased consumption of caffeine-enriched foods, beverages, and medicines worldwide (10, 11).

Increasing evidences suggest that caffeine concentrations exert adverse impacts on aquatic and terrestrial species (11, 12). For example, several studies reported the distribution of caffeine in tissues of aquatic organisms including macroalgae, fishes, clams, and other aquatic plants after being grown in the caffeine-contaminated environment (11–16). Studies have also reported caffeine accumulation in the coastal ecosystems, raising concerns about its potential impacts on the ecological safety (12–16). Additionally, many studies have shown that caffeine and its downstream metabolites is involved in human diseases (17, 18). For example, caffeine is involved in the development of colorectal cancer pathogenesis, metastasis, and prognosis (17). Adverse impacts of caffeine include induced oxidative stress and lipid peroxidation, influencing energy reserves and metabolic activity, changing reproduction and development, and neurotoxic effects (11, 12). Thus, caffeine has been considered as one of the most representative pollutants among pharmaceutically active compounds due to its high abundance in the environment (11, 12). Furthermore, since the human body loses active urate oxidase (UOX) in caffeine metabolism pathway, uric acid must be excreted without utilization or decomposition after caffeine intake (18). Increased uric acid production and decreased uric acid excretion in the human blood result in an abnormally high level of uric acid, which leads to the development of hyperuricemia symptoms, eventually developing into gout (18).

Considering the increasing impacts of caffeine pollution to the natural environment and human health, there is an urgent need to develop processes to remove caffeine, either by yielding decaffeinated products or by degrading environmental caffeine (7, 10–12, 17, 18). Traditionally, the level of caffeine in plants is decreased due to low activity of caffeine biosynthetic genes or the rapid degradation of caffeine (19, 20). In tea and coffee plants, caffeine is slowly catabolized by the removal of three methyl groups, resulting in the formation of xanthine (7, 21, 22). Xanthine is further degraded by the conventional purine catabolism pathway and finally results in CO_2_ and NH_3_ release (7, 21, 22). A recent study indicated that the conversion of caffeine to theacrine by C8 oxidation and followed with N9 methylation by N9-methyltransferase (20).

Several species of caffeine-degrading microbes have been isolated, including *Acetibacter* sp., *Acinetobacter* sp., *Alcaligenes fecalis*, *Aspergillus tamarii*, *Flavobacterium* sp., *Klebsiella, Moraxella* sp., *Pseudomonas* sp., *P. alcaligenes*, *P. putida*, *P.* s*cepacia*, *Serratia marcescens*, *Rhodococcus*, and *Trichosporon asahii* (10). Vilanova et al. analyzed the coffee-machine bacteriome and revealed a significant bacterial diversity in many identified genera, suggesting a potential driver of biotechnologically relevant processes, including decaffeination and bioremediation (23). However, the caffeine-degrading microbes in tea fields have not been well observed, which may have coevolved with tea plants to degrade caffeine and related purine alkaloids (4, 5). The treatment of coffee and tea wastes with purine alkaloids-degrading microorganisms may transform waste into valuable nutrients or byproducts thereby improving food quality and food safety (7, 10, 18). For instance, *Arxula adeninivorans* urate oxidase was recently reported to have high activity in reducing uric acid in beer, beef, and yeast extracts suggesting its potential role in low purine food production (18).

In this study, we performed transcriptomics using Illumina next-generation sequencing technology on *C. camelliae*, which was isolated from a tea field during its interaction with tea leaves. We compared the sequence data derived from the fungi to understand the mechanisms by which they catalyze caffeine and promote plant disease development. To study this, we generated *C. camelliae* urate oxidase (*CcUOX*) mutants and overexpressed *CcUOX* gene in *Escherichia coli* BL21. Urate oxidase is involved in the caffeine metabolism pathway by degradation of uric acid. Our findings revealed *CcUOX* mutants reduced fungal virulence, affected host defense response, and impaired uric acid degradation *in vivo*. The recombinant CcUOX protein efficiently degraded uric acid *in vitro*, thereby indicating a promising application in food with low purine and uric acid contents.

## 2. Materials and methods

### 2.1. Plant and fungi cultivation and treatment

*Camellia sinensis* cultivar Longjing 43 from Hangzhou, Zhejiang Province was used in this study. Healthy 2-year-old plants were placed in a disease-free climate chamber (PGX-600C, Saifu, China) under 12 h/12 h dark/light conditions (3). Fresh, mature leaves were collected randomly for fungal inoculation. The *C. camelliae* strain CCA was originally isolated from tea cultivar Longjing 43 in Hangzhou, Zhejiang Province (3) and cultivated on potato dextrose broth (PDB) medium at 25°C for approximately 3 days. Spores were harvested and frozen at −80°C. For the inoculation of tea plants, spores were diluted in ddH_2_O to a final concentration of 10^6^ spores/ml. The infection was performed as previously described (3). Briefly, spores were incubated on mature tea leaves for 24 h and recovered for RNA sequencing. For the untreated control, spores were incubated with ddH_2_O. Incubation was performed in a climate chamber at 25°C. For qPCR analysis, spores were collected at 12 and 24 h. Three independent biological replicates were used in each experiment.

To determine the effect of caffeine on *C. camelliae* gene expression, the fungi were incubated on solid potato dextrose agar (PDA) medium. Caffeine (Aladdin, Shanghai, China) was mixed with sterile melting medium to obtain final concentrations of 10, 100, and 500 μg/ml. Each experiment was done in triplicate, and plates without caffeine (0 μg/ml) were used as non-caffeine-incubated controls. Fresh *C. camelliae* CCA spores were used as a 0-h control. All mycelia were harvested 3 days after treatment, during which they were frozen at −80°C for use in qPCR assays.

### 2.2. Library construction, RNA sequencing, and quantitative real-time PCR

RNA samples were collected for Illumina sequencing. RNA purification, monitoring, cDNA library construction, and sequencing was performed as previously described (LC-Bio Technology Co., Ltd., Hangzhou, China) (24). For qPCR, total RNA from *C. camelliae* was extracted using TRIzol™ Reagent following the manufacturer’s instructions (Invitrogen, USA). cDNA was observed using 2 μg DNase-treated RNA, primer, and Superscript III Polymerase (Invitrogen, USA) made up to a total volume of 20 μl. The cDNA was diluted 1:20 with water, and 2 μl of the diluted cDNA was mixed with SYBR Green Supermix (Takara, Dalian, China) for real-time PCR experiments (2). qPCR was performed as previously described according to the manufacturer’s instructions (Applied Biosystems™ 7500, USA) (24). Primer sequences are listed in Supplementary Table 1.

### 2.3. *De novo* transcript assembly, gene annotation and functional classification

Cutadapt (version 1.9) and in-house Perl scripts were used to remove the reads containing (i) adaptor contamination, (ii) low quality bases, and (iii) undetermined bases. The quality of the sequences was verified by FastQC (version 0.10.1), including Q20, Q30, and GC content of clean data (24). *De novo* assembly was performed using Trinity (version 2.4.0) (25). The raw sequence data has been submitted to the NCBI Short Read Archive with accession number GSE205689. All assembled unigenes were aligned against the following databases using DIAMOND (version 0.7.12) with a threshold of E < 0.00001. These included the non-redundant (Nr) protein database,^1^ SwissProt,^2^ the Gene Ontology (GO),^3^ the Kyoto Encyclopedia of Genes and Genomes (KEGG),^4^ and eggNOG.^5^ Finally, the FPKM of each gene was calculated based on the length and reads count.

### 2.4. Analysis of differentially expressed genes

Differentially expressed genes (DEGs) in all samples (control: CCK and CT24h: *C. camelliae* infected tea plant for 24 h) were analyzed as previously described (24). DEGs that were selected had a log2 (fold change) > 1 or a log2 (fold change) < −1 and were statistically significant (*p* < 0.05) using edgeR (R package, version 3.12.1) (26). Next, GO and KEGG enrichment analyses were performed based on the differentially expressed unigenes using in-house Perl scripts. GO enrichment analysis of DEGs was carried out by the GOseq R package, wherein gene length bias was corrected (27). KEGG is a database based on large scale molecular datasets generated by genome sequencing and other high-throughput experimental technologies (28).

### 2.5. Strain construction

The target gene replacement vector was first generated by PCR-amplifying the flanking sequences of the *CcUOX* gene, using *C. camelliae* CCA genomic DNA as a template. The fragments were then inserted into the replacement vector PXEH (24). *Agrobacterium tumefaciens* strain AGL1, which contained a recombinant replacement vector, was transformed into *C. camelliae* CCA spores. Knockout strains were screened on selective media, as confirmed by PCR and qPCR. To generate *CcUOX* gene complement lines, the full-length gene was amplified and transformed into Δ*CcUOX* mutants. The strains showing wild-type gene expression levels were used for further analysis.

### 2.6. Antifungal function analysis of caffeine

The antifungal activity of caffeine toward *C. camelliae* was assessed by measuring mycelial plug growth. Caffeine was premixed with sterile melting PDA medium to obtain a final concentration of 500 μg/ml. Fungi were incubated and mycelial growth was measured and compared at different time. Each treatment was performed in triplicate, and PDA plates containing distilled water were used as the control. The incubation of fungi, measurement of mycelial growth, and relative inhibition ratio were evaluated as described previously (3, 4). Briefly, the relative inhibition ratio was calculated by using the following formula: *I* (%) = [(*C* − *d*) − (*T* − *d*)]/(*C* − *d*) × 100, where *I* (%) is the inhibition rate, *d* is the diameter of the mycelial plug disk (6 mm), and *C* and *T* are the colony diameters of the control and treatment, respectively.

### 2.7. *CcUOX* overexpression, purification, characterization, and degradation of uric acid

Full length cDNA of the *CcUOX* gene was cloned into the expression vector pET28a to construct the plasmid pET28a-*CcUOX* with His-tag. The plasmid was then transformed into competent cells of *Escherichia coli* BL21. The recombinant protein was induced and purified by affinity chromatography on nickel Ni-NTA resin (Sangon, China). The purified proteins were determined by using 10% sodium dodecyl sulfate-polyacrylamide gel electrophoresis (SDS-PAGE). The enzyme activity test of CcUOX was carried out based on the spectrophotometric determination of the substrate of uric acid according to previous study (18). The effect of pH on CcUOX activity was evaluated in buffers ranging from pH 2.0 to 8.0 (2). The effect of temperature on UOX activity was investigated at temperatures ranging from 0 to 80°C.

### 2.8. Statistical analysis

Analysis of variance was performed using the statistical product and service solutions 18 software (IBM, New York, NY, USA). The differences were considered significant at *p < 0.05, **p < 0.01, and ***p < 0.001, respectively. All data are represented as mean ± SEM of 3 independent replicates.

## 3. Results

### 3.1. Identification and characterization of differentially expressed genes in *C. camelliae* during interaction with tea plant

To identify the key factors involved in *C. camelliae* incubation in tea plants, we first performed RNA-sequencing. Previous work has indicated that the tea plant cultivar Longjing 43 (LJ43) is compatible with *C. camelliae* (3, 5). At 24 h post-infection (hpi), appressoria were observed and the fungi remained on the surface of the tea leaves. Therefore, samples were collected 24 h after the interaction of *C. camelliae* with the tea plant cultivar LJ43. *C. camelliae* spores incubated with ddH_2_O were used as control.

cDNA from *C. camelliae* was isolated and sequenced on an Illumina High-Seq 2500 platform using paired-end sequencing. The unincubated control samples were named CCK_1, CCK_2, CCK_3, and the *C. camelliae* samples that had interacted with tea plant for 24 h were named CT24_1, CT24_2, and CT24_3. A total of 310,666,862 raw reads representing 46.8 Gb were obtained (Supplementary Table 2). After removing low-quality reads, approximately 304,358,620 (98.0% of the raw reads) clean reads, representing 44.8 Gb, were observed (Supplementary Table 3). All valid reads from the six RNA-seq datasets were used for transcript assembly. A total of 26,138 unique genes were identified. The length of transcripts ranged from 201 to 16,301 bp, with an average length about 1,175 bp.

**FIGURE 1:**
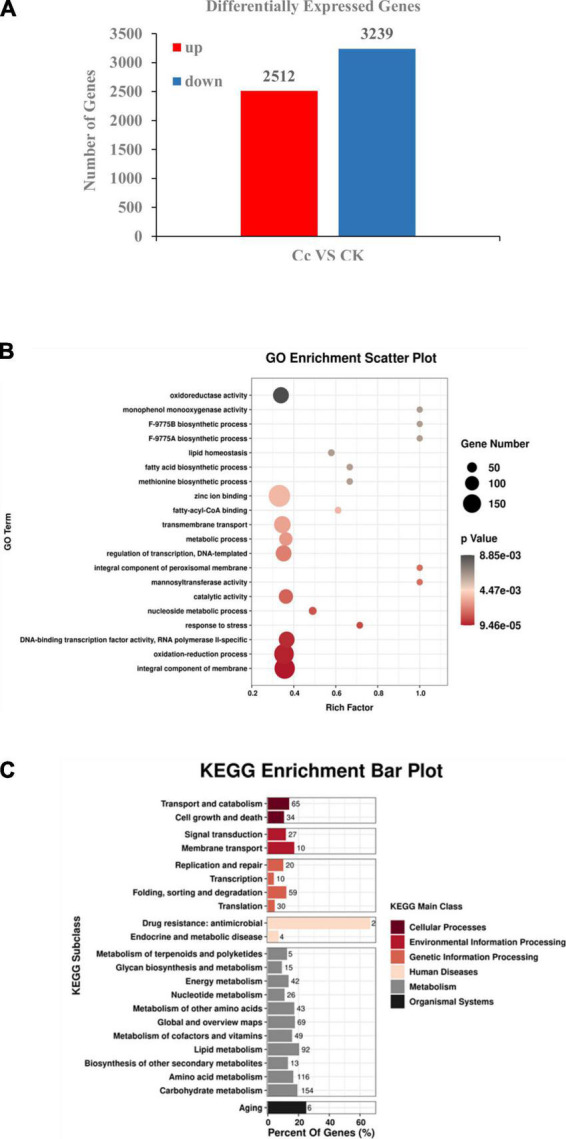
Transcriptomic analysis revealed differentially expressed genes in *C. camelliae* CCA during infection tea plant. **(A)** Numbers of differentially expressed genes (≥2-fold; *p* ≤ 0.05) in *C. camelliae* at 24 h after interaction with tea plant (Cc) or without interaction with tea plants (CK) by RNA-seq. **(B)** GO analysis of differentially expressed genes in *C. camelliae* during infection tea plant. **(C)** KEGG analysis of differentially expressed genes in *C. camelliae* during infection tea plant.

BLAST was then performed to identify the transcripts of other organisms homologous to the unique assembled genes of *C. camelliae*. The NCBI non-redundant (NR) protein database, KEGG, GO, eggNOG, Pfam, and Swissprot databases were included for functional annotation of *C. camelliae*. Finally, data from the treatments (CK and CT24h) and biological replicates allowed us to identify DEGs in *C. camelliae*. At 24 hpi, the expression of 5,751 genes in *C. camelliae* was Significantly Statistically altered by Two-Fold or more (SSTF, *p* ≤ 0.05) when compared to water-incubated spores, with 2,512 genes (44%) being upregulated and 3,239 genes (56%) being downregulated (Figure 1A).

Gene Ontology enrichment analysis revealed functional terms that were significantly enriched at the genome level. GO analysis to characterize SSTF genes revealed the enrichment of terms related to transcription regulation, DNA-template, DNA-binding transcription factor activity, RNA polymerase II, transmembrane transport, oxidation-reduction, stress response, oxidoreductase activity, and metabolism when compared to the whole genome (*p* < 0.05) (Figure 1B). These results indicate the direct involvement of the above processes in *C. camelliae* interaction with tea plant. The SSTF genes were then mapped to KEGG reference pathways (Figure 1C). Several pathways associated with pathogenicity and metabolism were enriched, including transport and catabolism, membrane transport, transcription, and metabolism of various molecules, such as nucleotides, lipids, amino acids, and carbohydrates.

### 3.2. Differential expression of transporters during *C. camelliae* interaction with tea plant

Under natural conditions, microorganisms encounter multiple natural toxic chemicals, either from other competent organisms or from the environments. Microbes have systems that can transport toxic chemicals to the external environment. Transporters in the major facilitator superfamily (MFS) and ATP-binding cassette (ABC) family play major roles in transport processes (29–31). ABC transporters are regarded as primary active transporter systems (29). ABC proteins are found in all living cells and are often involved in multidrug resistance of microbial pathogens. ABC transporters use ATP as an energy source to hydrolyze nucleotide triphosphates and mediate membrane transport. MFS transporters are secondary active transport systems that are unable to hydrolyze ATP and are membrane transporters that function as uniporters, symporters, or antiporters (30).

**FIGURE 2:**
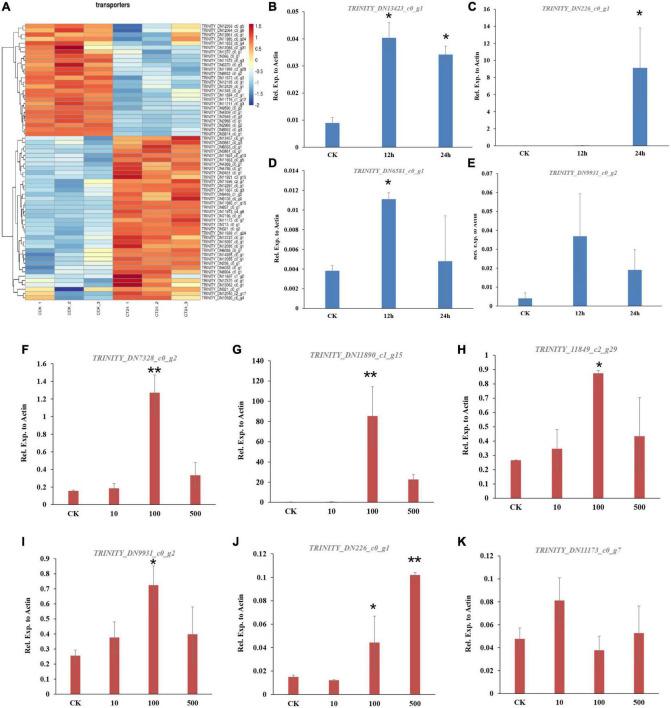
Genes encoding transporters are enriched in *C. camelliae* CCA during infection tea plant. **(A)** Heatmap of differentially expressed genes encoding transporters. **(B–E)** qPCR analysis of selected transporter genes in *C. camelliae* during infection tea plant. **(F–K)** PCR analysis of selected transporter genes in *C. camelliae* upon caffeine treatments. Different concentration of caffeine (CK: 0, 10, 100, and 500 μg/ml) was used to treat *C. camelliae.* All data were normalized to the expression of *CcActin*. Error bars represent SD of three biological replicates. Asterisks indicate significant differences between treatment and CK (*p < 0.05; **p < 0.01).

To reveal the global patterns of transporter genes in *C. camelliae* during interaction with tea plant, the GO term of the transporter was analyzed. We found that sixteen ABC transporter genes and forty-eight MFS genes were significantly differentially expressed during this interaction (Figure 2A and Supplementary Table 4). Forty of them were upregulated, while twenty-four were downregulated. Several of these transporters were confirmed using qPCR analysis. The genes *DN13423_c0_g1*, *DN9931_c0_g2*, and *DN3418_c0_g3* were induced at both 12 and 24 h (Figures 2B, E and Supplementary Figure 1A). However, the expression of *DN6581_c0_g1* and *DN12164_c0_g1* was increased at 12 h, whereas the induction of *DN226_c0_g1* was only observed at 24 h (Figures 2C, D and Supplementary Figure 1B). Transporter-related DEGs thus have diverse roles during *C. camelliae* interaction with tea plant.

Since caffeine is toxic to certain microbes, we next tested whether *C. camelliae* transporter genes were involved and induced by caffeine. The fungi were incubated with different concentrations of caffeine (10, 100, or 500 μg/ml) for 72 h. The expression of *DN226_c0_g1*, *DN11890_c1_g15*, *DN11849_c2_g29*, *DN7328_c0_g2*, *DN9931_c0_g2*, and *DN11173_c0_g7* increased in the presence of caffeine (Figures 2F–K). These results indicate that transporter genes may be involved in caffeine transport in *C. camelliae*, which might contribute to fungal virulence.

### 3.3. *C. camelliae* urate oxidase is involved in the caffeine degradation pathway and contributes to fungal virulence

**FIGURE 3:**
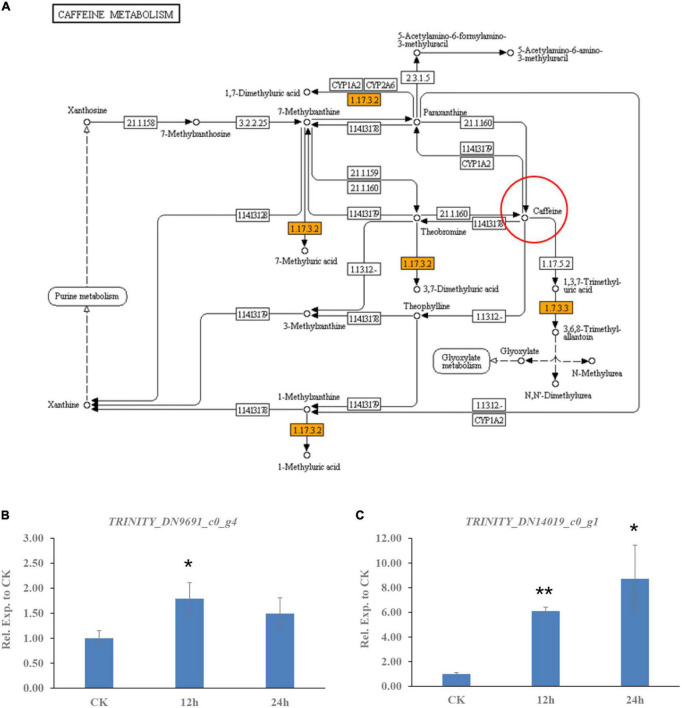
Caffeine metabolism pathway genes involved in *C. camelliae* CCA interaction with tea plant. **(A)** KEGG map of differentially expressed genes in caffeine metabolism pathway. **(B)** Expression of gene encoding xanthine dehydrogenase (XDH) was induced in *C. camelliae* CCA during interaction with tea plant. **(C)** Expression of gene encoding urate oxidase (CcUOX) was increased in *C. camelliae* CCA during interaction with tea plant.

**FIGURE 4:**
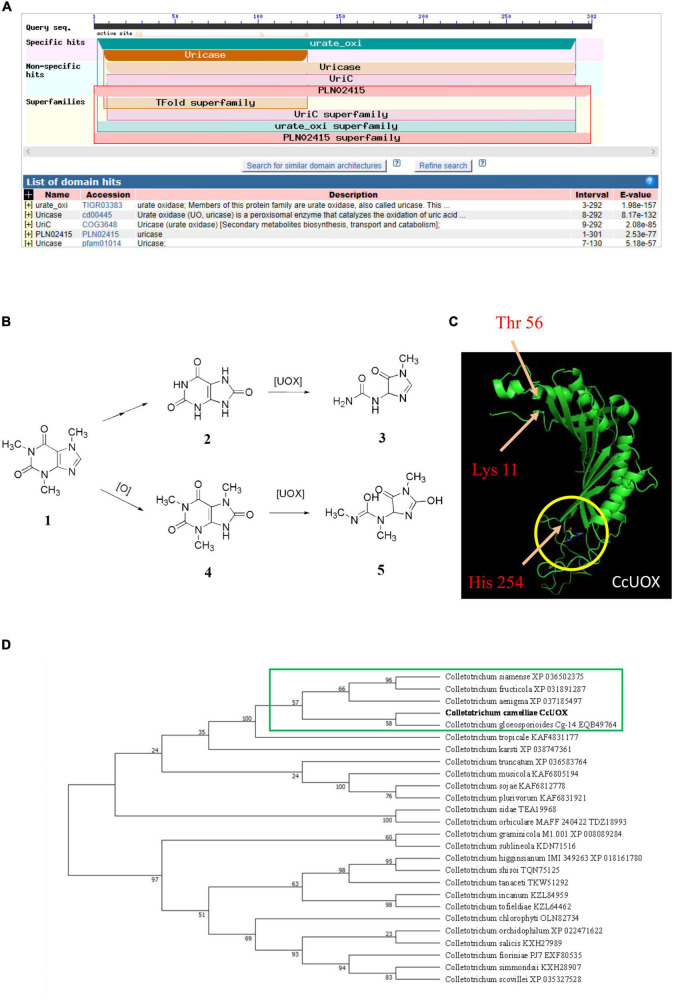
Characteristics analysis of CcUOX in *C. camelliae.* **(A)** Blast analysis indicates CcUOX protein contained conserved domains of urate oxidase. **(B)** UOX involved in caffeine metabolism. 1. Caffeine, 2. Uric acid, 3. Allantoin, 4. 1,3,7-trimethyluric acid, 5. 3,6,8-trimethylallantoin. **(C)** The predicted structures of CcUOX. The arrows indicated the conserved catalytic triad residues. The circles indicated the predicted ligand binding sites. **(D)** Phylogenetic tree of CcUOX proteins with indicated *Colletotrichum* species. The indicated proteins including *Colletotrichum gloeosporioides* Cg-14 (EQB49764), *C. siamense* (XP 036502375), *C. fructicola* (XP 031891287), *C. aenigma* (XP 037185497), *C. tropicale* (KAF4831177), *C. karsti* (XP 038747361), *C. truncatum* (XP 036583764), *C. musicola* (KAF6805194), *C. sojae* (KAF6812778), *C. plurivorum* (KAF6831921), *C. sidae* (TEA19968), *C. orbiculare* MAFF 240422 (TDZ18993), *C. graminicola* M1.001 (XP 008089284), *C. sublineola* (KDN71516), *C. higginsianum* IMI 349263 (XP 018161780), *C. shisoi* (TQN75125), *C. tanaceti* (TKW51292), *C. incanum* (KZL84959), *C. tofieldiae* (KZL64462), *C. chlorophyti* (OLN82734), *C. orchidophilum* (XP 022471622), *C. salicis* (KXH27989), *C. fioriniae* PJ7 (EXF80535), *C. simmondsii* (KXH28907), *C. scovillei* (XP 035327528).

In addition to transporters, the degradation or catabolism of toxic compounds could be a mechanism for *C. camelliae* virulence. While screening the transcriptome of *C. camelliae* during interaction with tea plant, two genes, *DN9691_c0_g4* and *DN14019_c0_g1*, which are involved in caffeine metabolism, were observed by KEGG analysis (Figure 3A). *DN9691_c0_g4* encodes xanthine dehydrogenase (XDH), whereas *DN14019_c0_g1* encodes a gene mapped to the hypothetical protein CGLO_10871 in *C. gloeosporioides* Cg-14. The expression of both genes increased at 12 h and 24 h (Figures 3B, C). BLAST analysis indicated that *DN14019_c0_g1* encodes a protein containing the conserved urate oxidase domain (Figure 4A). Urate oxidase (EC 1.7.3.3) is a major enzyme in caffeine degradation that catalyzes the oxidation of uric acid to allantoin and hydrogen peroxide, using oxygen as an electron receptor (Figure 4B) (18). Other reports have indicated that UOXs are involved in the C-8 oxidation pathway of caffeine (32–34).

A 906-bp full-length gene containing an entire open reading frame (ORF) was then cloned from CCA using PCR. The ORF encoded a 301-AA protein with a predicted molecular weight of 33.8 kDa. BLAST analysis of the protein sequence showed the highest sequence identity with previously characterized UOXs, and was named *CcUOX*. Phylogenetic analysis indicated that *CcUOX* is highly similar to UOXs from its neighbors in *Colletotrichum* spp. (Figure 4D). The active site residues of UOXs were well conserved in CcUOX, based on multiple sequence alignment analysis with previously reported UOXs (18). These include: (i) Thr 56, Arg 175, Gln 226, and Asn 252, which hold the substrate like molecular tweezers; (ii) Phe 158 closing one end of the cavity below; and (iii) the catalytic triad residues Lys 11, Thr 56, and His 254.

The protein structure of CcUOX was predicted using Iterative Threading ASSEmbly Refinement (I-TASSER) (35). The structure contained at least five helices, separated into two clusters by at least seven strands (Figure 4C). The predicted helices and strands provide a complex skeleton for UOX activity. The catalytic residues Lys, Thr, and His were present in the predicted CcUOX structure with positions similar to those in other UOXs (Figure 4C) (18). The first two residues were located at the N-terminus of the protein, whereas the His residue was located at the C-terminus (Figure 4C). It has been reported that UOXs form a tetramer composed of two dimers stacked face-to-face and reorganize in a crystallographic 2-fold axis (36). In this structure, all the active sites are located at the interface between the two monomers in a cavity exposed to the solvent (18, 36). Under these conditions, Lys and Thr residues are present in one monomer, while His, Arg, Gln, and Asn are located in another monomer (18, 36). Here, all the conserved residues in CcUOX suggest a possible role in enzyme activity and might interact with each other via a similar mechanism. Since CcUOX is involved in the caffeine metabolism pathway, we hypothesized that CcUOX plays a role in disease development.

**FIGURE 5:**
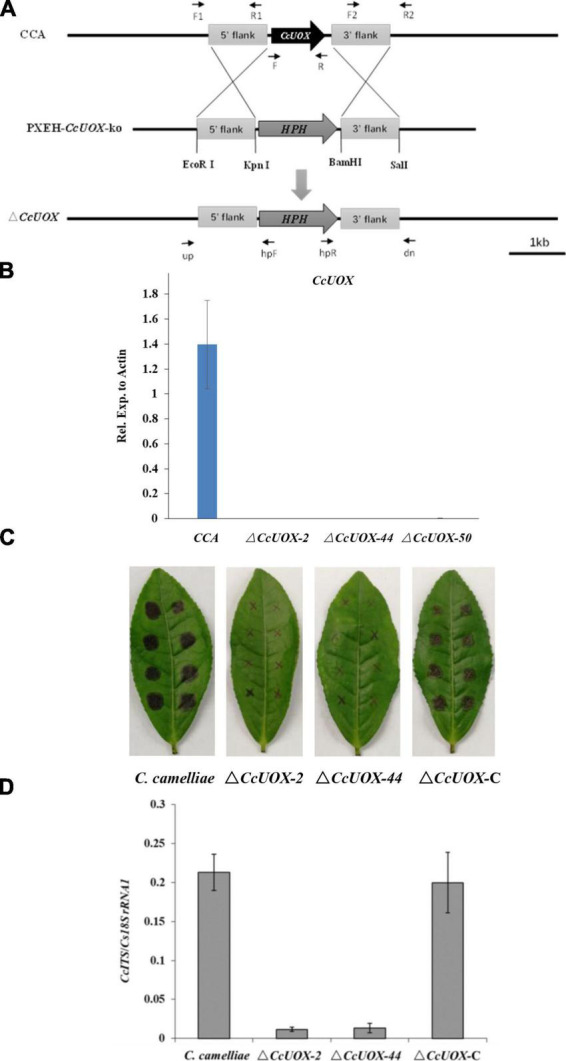
*C. camelliae CcUOX* involves in fungal virulence. **(A)** Strategy for construction of *CcUOX* gene deletion mutants. **(B)** qPCR method conformation of Δ*CcUOX* mutants. **(C)** Deficient of *CcUOX* in *C. camelliae* reduced fungal virulence toward tea plant cultivar LJ43. **(D)** Fungal biomass indicated less pathogen was observed in Δ*CcUOX* mutants infected plants compared with wild-type CCA and the compliment lines Δ*CcUOX-*C-*CcUOX*.

To determine the role of CcUOX, we deleted its gene using the gene replacement method (Figure 5A). The putative Δ*CcUOX* mutants were verified by PCR using gene-specific primers, and no bands were detected (Supplementary Figure 3). The mutants were further confirmed by qPCR analysis, and no gene expression was observed when compared to the wild-type strain (Figure 5B). This indicates that *CcUOX* was successfully deleted in the mutants. In addition, the complement strain Δ*CcUOX-*C*-CcUOX* was constructed by reintroducing the *CcUOX* gene into the mutant strain, which was confirmed by RT-PCR analysis (Supplementary Figure 4).

Wild-type CCA, Δ*CcUOX* mutants, and Δ*CcUOX-*C*-CcUOX* were incubated on tea leaves for 3 days. The lesion size was much smaller in the Δ*CcUOX*-mutant-infected tea plant (Figure 5C) than in the wild-type and complemented lines. Fungal growth was then quantified by qPCR, and fewer fungi were detected in the mutants that infected tea plants (Figure 5D). These results indicate that Δ*CcUOX* is less virulent toward tea plant and that *CcUOX* is associated with pathogenicity in *C. camelliae*.

### 3.4. Tea caffeine inhibited *C. camelliae* mycelial growth and CcUOX involved in reducing uric acid content

Tea is rich in alkaloids and flavonoids such as caffeine and catechins, which play key roles in defense. Since biotic stress can induce caffeine accumulation, plants may use endogenous caffeine to resist pathogens (37). Recent reports indicate that caffeine can inhibit the growth of tea pathogens, including the virulent fungus *C. camelliae* and the less virulent pathogen *C. fructicola* (4, 5). The inhibition rate of caffeine is higher in *C. fructicola* than in *C. camelliae* (4). This indicates that *C. camelliae* was more tolerant to caffeine, whereas *C. fructicola* was more vulnerable, suggesting that the former evolved ways to overcome caffeine-mediated plant defenses.

**FIGURE 6:**
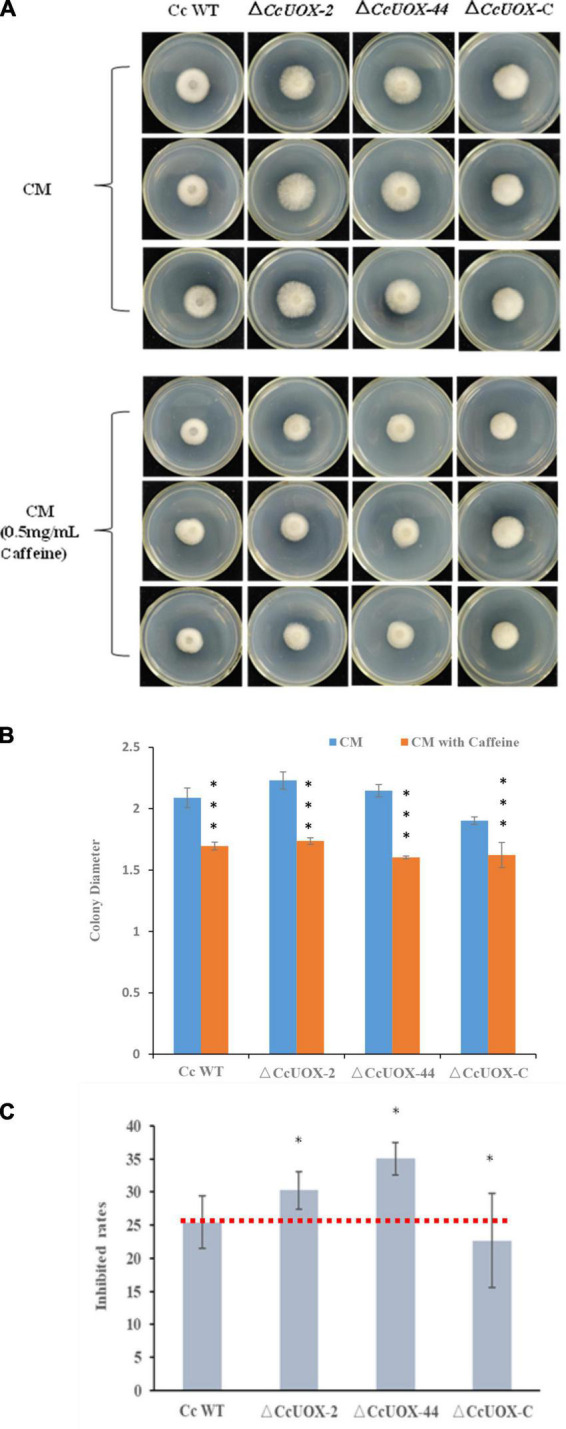
*C. camelliae CcUOX* involved in fungal tolerance toward caffeine. **(A)** Caffeine inhibited *C. camelliae* wild-type CCA and Δ*CcUOX* mutant growth. **(B)** Colony diameters of *C. camelliae* wild-type CCA, Δ*CcUOX* mutants and Δ*CcUOX-*C-*CcUOX* upon caffeine treatments. Error bars represent SD of three biological replicates. Asterisks indicate significant differences between treatment (CM with caffeine) and un-treatment (CM) (***p < 0.001). **(C)** The inhibition rates of caffeine toward *C. camelliae* wild-type CCA, Δ*CcUOX* mutants and Δ*CcUOX-*C-*CcUOX*. Error bars represent SD of three biological replicates. Asterisks indicate significant differences between *C. camelliae* indicated strains and *C. camelliae* wild-type CCA (*p < 0.05).

Since caffeine has antifungal activity and *CcUOX* is involved in caffeine metabolism pathway, we further investigated whether the mutant exhibited reduced pathogenicity due to vulnerability to caffeine. When compared to the control, caffeine (500 μg/ml) inhibited *C. camelliae* growth, and the inhibition rate was approximately 25% at 2 days post-infection (dpi) (Figures 6A–C). It indicated *C. camelliae* CCA was tolerant to caffeine at this concentration. However, the inhibition rate was higher in the Δ*CcUOX* mutants (30–35%), indicating that the mutants were more vulnerable to caffeine than the wild-type CCA (Figure 6C). The complement lines restored wild-type tolerance toward caffeine (Figures 6A–C). Based on these results, we concluded that *CcUOX* contributes to the tolerance of *C. camelliae* CCA to caffeine.

**FIGURE 7:**
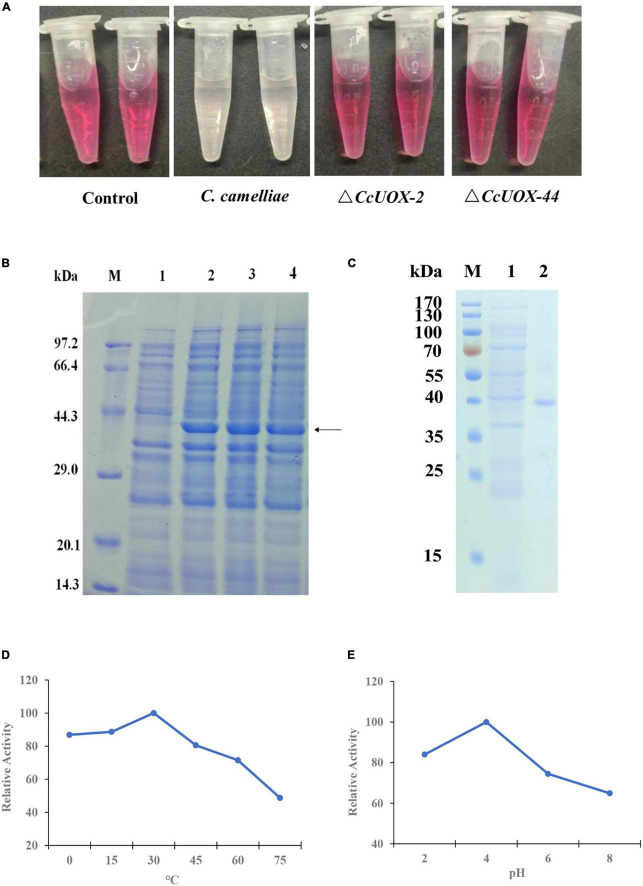
CcUOX involved in uric acid degradation both *in vivo* and *in vitro*. **(A)** *C. camelliae* CcUOX involved in uric acid degradation *in vivo*. In the control sample, no fungi were added and the uric acid was not degraded at all. In the *C. camelliae* wild-type strain CCA added sample, the uric acid was almost lost as the color was clear. In the Δ*CcUOX* mutants incubated samples, the uric acid was not degraded as the color was not changed since the deletion of *CcUOX*. **(B)** SDS-PAGE analysis of recombinant CcUOX in *Escherichia coli*. The proteins were stained with Coomassie brilliant blue R-250. M, Position of marker proteins. Line 1, SDS-PAGE of the extract from empty pET28a-transformed bacterial cultures (control). Line 2–4, SDS-PAGE of the recombinant CcUOX protein which was induced from pET28a-CcUOX-transformed different bacterial cultures. The arrow indicates the recombinant CcUOX protein. **(C)** SDS-PAGE of the recombinant CcUOX protein. M, Position of marker proteins. Line 1, SDS-PAGE of the extract from control bacterial culture. Line 2, the partly purified CcUOX protein. **(D,E)** The optimum temperature **(D)** and pH **(E)** for recombinant CcUOX protein in catalyzing uric acid *in vitro*. The highest activity was considered as 100% and all the experiments were replicated thrice with similar patterns.

Next, we tested whether CcUOX is involved in catalyzing uric acid. Wild-type CCA and Δ*CcUOX* mutants were used to degrade uric acid. Since UOXs catalyze the oxidation of uric acid to produce hydrogen peroxide, we tested the content of uric acid based on the change in hydrogen peroxide. When compared to the control sample (0.1% uric acid), the color of CCA-treated samples (0.1% uric acid plus spores of CCA) almost disappeared after being incubated for 48 h, while the color of Δ*CcUOX*-mutant-treated samples (0.1% uric acid plus spores of Δ*CcUOX* mutants) did not change (Figure 7A). These results indicate that the loss of CcUOX significantly reduced the ability of the fungus to degrade uric acid *in vivo*.

To characterize the function of *CcUOX*, we generated an expression vector containing the *CcUOX* gene into the plasmid pET28a and transformed it into *Escherichia coli* BL21 to overexpress the protein. Four hours after induction, the recombinant *E. coli* BL21 strains contained pET28a-*CcUOX* produced the indicated CcUOX proteins while the *E. coli* BL21 contained an empty vector not produced (Figure 7B). The molecular weight of the recombinant CcUOX protein was around 40 kDa based on the SDS-PAGE analysis, which is a bit higher than the predicted molecular weight (33.8 kDa). The higher molecular weight may be because of the vector pET28a which contained the HA-tag and multiple cloning site sequence (MCS). Next, we identified the recombinant CcUOX protein from *E. coli* BL21 and partly purified the protein by Ni-NTA chromatography (Figure 7C). The recombinant CcUOX protein showed an optimal temperature activity-relationship with maximum activity at 30°C and over 80% of the maximum activity at temperatures between 0 and 30°C (Figure 7D). The optimal pH value of the CcUOX protein activity was about 4.0, and >80% of the maximal activity was retained at pH values between 2.0 and 4.0 (Figure 7E). These results indicate that the recombinant CcUOX protein could reduce uric acid content *in vitro*. A similar result was observed in *A. adeninivorans* when rUOX was involved in reducing uric acid *in vitro* (18).

## 4. Discussion

Plant defense compounds have broad antifungal activity, and are constitutively synthesized in plant tissues or induced by microbes (38). This category includes all the compounds classified as phytoalexins and phytoanticipins. In tea field, fresh tea leaves and processed tea materials contain a large number of metabolites that can be divided into volatile and non-volatile aromatic compounds (1). The non-volatile components of tea include polyphenols, carbohydrates, amino acids, organic acids, flavonols, vitamins, caffeine, and purine derivatives (1). They are key compounds that affect tea taste, determine the color of tea juice, and have beneficial effects on humans (1).

Some tea compounds exhibit antimicrobial activity. For example, the contents of total phenolics, catechins, and caffeine were compared in two tea cultivars with different resistance to the microbe *C. fructicola* (8, 39). Caffeine, eougakkicatecub-3-gallate (EGCG), and catechin were induced in the resistant tea cultivars. *In vitro* antifungal activity tests have shown that caffeine strongly inhibits mycelial growth (8). Further studies revealed that genes associated with phenylpropanoids and flavonoids were also enriched in the resistant tea cultivar, suggesting that caffeine and flavonoid biosynthesis are correlated with tea plant defense (39). Other reports have indicated that certain secondary metabolites have antimicrobial activity (40). For instance, quercetin and cyanidin aglycones can inhibit hyphal growth and conidial germination in *C. gloeosporioides* (41, 42). It has also been speculated, based on microarray data, that tea plant resistance to *C. camelliae* may be associated with the phenylpropanoid and flavonoid pathways (5). Our data revealed that caffeine significantly inhibited *C. camelliae* CCA growth. The inhibition rate increased at higher caffeine concentrations, similar to previous reports (4).

The antimicrobial activity of caffeine may be different among *Colletotrichum* species. Lu et al. compared the differences in the pathogenicity of *Colletotrichum* spp. and observed that *C. camelliae* LS-19 was more virulent than *C. fructicola* SX-6 (4). Interestingly, when tea compounds were detected during mycelial growth *in vitro*, *C. fructicola* SX-6 was more vulnerable to caffeine and catechins than *C. camelliae* LS-19. Therefore, different *Colletotrichum* species have different tolerances to caffeine, with *C. camelliae* being more tolerant, and *C. fructicola* being more vulnerable. Since these compounds are involved in plant defense, successfully infected microbes have evolved the ability to overcome host defenses. Here, we revealed that *C. camelliae* CCA is a caffeine-tolerant strain with the ability to escape or detoxify caffeine.

The growing belief that the ingestion of caffeine has adverse effects on human health has resulted in an increasing demand for methods to remove caffeine, either to degrade environmental caffeine or to yield decaffeinated metabolites (10, 21). In the major caffeine degradation pathway in coffee and tea plant, exploration of the isotopic markers of caffeine has revealed the following pathway: “caffeine → theobromine/theophylline → 3-methyxanthine → xanthine → uric acid → allantoin → allantoic acid → CO_2_ + NH_3_” (22, 43). However, other pathways that degrade caffeine may also exist in *Camellia* plants (34). Therefore, the identification of genes in the caffeine metabolism pathway is of considerable value in research related to caffeine degradation in *Camellia* plants.

Caffeine metabolic pathways have also been reported in some microorganisms (32, 44). Bio-decaffeination methods are promising due to their specificity, eco-friendliness, and cost-effectiveness (10). *Pseudomonas* is well known for its ability to use caffeine as the sole source of carbon and nitrogen. In *Pseudomonas*, N-demethylation and oxidation pathways are involved in caffeine catabolism (10, 32). In the oxidation pathway, caffeine is directly oxidized at the C8 position to form 1,3,7-trimethyluic acid, which is further oxidized to 3,6,8-trimethylallantoin (10, 32). This pathway has been observed in both bacterial isolates and mixed cultures (33, 44, 45). In addition, genes associated with caffeine oxidation have been identified in *Pseudomonas* sp. strain CBB1 (10, 33). Recently, the caffeine-resistant bacterium *P. putida* CT25 was isolated from tea soil, and can survive in a medium with high caffeine content (32). Less is known about caffeine degradation in fungi than in bacteria. Previous studies have tested the ability of filamentous fungi to grow on caffeine as a sole source of nitrogen and found that *Penicillium* and *Aspergillus* were able to degrade the alkaloid with theophylline as the first degradation product (22).

Urate oxidase is involved in caffeine catabolism in both plants and microorganisms (22, 34). UOX is a major enzyme involved in purine metabolism (12, 22). UOXs are widely distributed in organisms, and microbe-derived UOXs are well characterized for their stable enzyme activity, wild source, and easy production expansion (18). Numerous microbial UOX genes have been identified, some of which have been exploited for this application (18). In this study, *C. camelliae* CCA was originally isolated from a tea field where it had colonized tea plant; the coevolution of these organisms suggests that special mechanisms may exist between *C. camelliae* and tea plant. To colonize tea plant, fungi may induce cell death and compromise the integrity of plant tissues during infection, which must actively detoxify host antimicrobials, such as caffeine. Detoxification is facilitated by various mechanisms, including metabolization of the compounds to the less toxic derivatives and transporter-mediated efflux to maintain plant defense compounds at sublethal thresholds (46). Here, we show that *CcUOX*, which encodes urate oxidase, is involved in caffeine metabolism pathway and plays a key role in uric acid degradation both *in vivo* and *in vitro*. Δ*CcUOX* mutants were more sensitive to caffeine, while the complement lines that reintroduced *CcUOX* not only enhanced the resistance to caffeine, but also restored wild-type virulence toward tea plant.

Other reports have also indicated that pathogens have detoxification mechanisms (46). For example, *Rhizoctonia solani* production of 5-hydroxycamalexin, *Botrytis cinerea* production of 3-indolecarboxylic acid, and *Sclerotinia sclerotiorum* glucosylation of camalexin have been observed as methods of avoiding camalexin toxicity (38). Since flavonoids have antifungal activity, the pathogens *S. sclerotiorum* and *B. cinerea* have developed a way to circumvent flavonoid defense using quercetin dioxygenases that catabolize flavonoids, such as quercetin and kaempferol (24, 47). Sakuranetin is a rice-flavanone-type phytoalexin. Rice fungi, such as *Pyricularia oryzae* and *R. solani*, can detoxify sakuranetin to compounds with much lower antifungal activity (48, 49). Benzoxazinoid (Bx) metabolites produced by wheat are active against *Fusarium* sp. (50). Deletion of the Bx detoxification gene *NAT1* from *F. graminearum* reduces deoxynivalenol production in spring wheat (50). Interestingly, *CcUOX* significantly degraded uric acid when incubated with *C. camelliae* CCA while the recombinant CcUOX protein efficiently reduced uric acid *in vitro*. Similarly, alkaline urate oxidase (AaUOX) from *A. adeninivorans* can efficiently reduce uric acid and purine content in beer, beef, and yeast extract (18).

In addition, during the interaction between tea plant and *C. camelliae*, the expression of many genes involved with transporters was observed. The large number of genes related to ABC transporters and the MFS suggest that they are involved in fungal colonization. These genes may be involved in the transport of toxic compounds, such as caffeine. When exposed to camalexin, *B. cinerea* induces the expression of the ABC transporter *BcatrB*, an efflux protein, which acts as a protective mechanism against the fungitoxic effect of camalexin (51). The MFS transporters *Bcmfs1* and *BcmfsG* from *B. cinerea* and *MgMfs1* from *Mycosphaerella graminicola* are required for protection against fungicides and natural toxic compounds (52, 53). *ChMfs1* is important for intra-hyphal hyphae formation and is involved in pathogenicity during the infection phases of *C. higginsianum* (25). Indeed, several genes encoding transporters were highly induced by caffeine treatment in our study. Such genes may be involved in caffeine transport. Further investigation is required to determine whether *C. camelliae* uses caffeine for nutrition.

## 5. Conclusion

Here, we compared the DEGs derived from the fungi to identify the genes used to degrade caffeine associated purine alkaloids and cause plant diseases. Transporters and several genes involved in caffeine metabolism pathway were enriched in *C. camelliae* during infection tea plant. We observed that a *CcUOX* gene (encoding urate oxidase) mutant alters fungal virulence and impairs fungal tolerance toward caffeine. *C. camelliae* efficiently reduced uric acid levels both *in vivo* and *in vitro*, based on its ability to degrade uric acid contents in Δ*CcUOX* mutants and recombinant CcUOX protein, indicating its potential application in reducing food with higher uric acid levels. Deepening our understanding of UOXs and other genes (e.g., *XDH*, Transporters, etc.) from tea field microbes involved in caffeine metabolism pathway are important for (i) the development of nutritional, low-purine foods suitable for patients with hyperuricemia and gout and (ii) for the reduction of caffeine-related contamination of soil and the surrounding environment in the future. Further studies should investigate the application of enzymatic degradation of purines and caffeine enriched food, beverage and/or the surrounding environment, particularly by recombinant CcUOX proteins.

## Data availability statement

The datasets presented in this study can be found in online repositories. The names of the repository/repositories and accession number(s) can be found in the article/Supplementary material.

## Author contributions

SL and XQ designed the research plan and wrote the manuscript. All authors performed the research and analyzed the data.

## Footnotes

1
http://www.ncbi.nlm.nih.gov/

2
http://www.expasy.ch/sprot/

3
http://www.geneontology.org

4
http://www.genome.jp/kegg/

5
http://eggnogdb.embl.de/
