# Supplementary material for: Urate oxidase from tea microbe Colletotrichum camelliae is involved in the caffeine metabolism pathway and plays a role in fungal virulence
Source: Front Nutr. 2023 Jan 4;9:1038806. doi: 10.3389/fnut.2022.1038806 (PMC9846643; doi:10.3389/fnut.2022.1038806)
Supplement: Supplementary file 1 [file Data_Sheet_1.pdf]

A

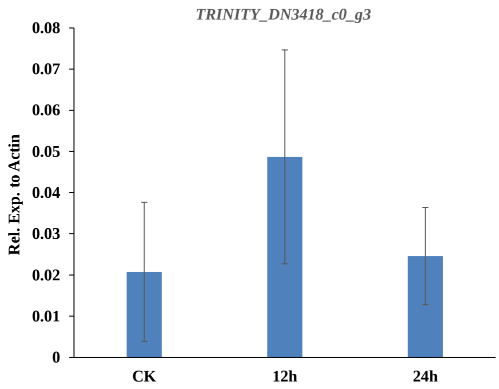

B

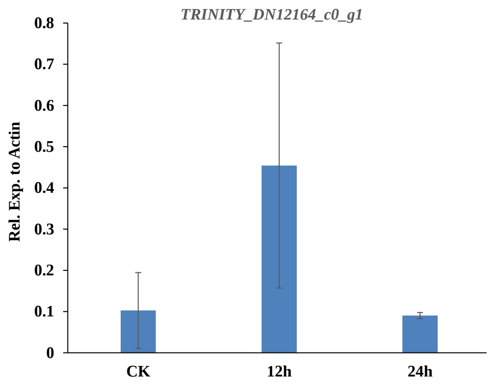

Figure S1. Differentially expressed genes in *C. camelliae* during infection tea plants.

A

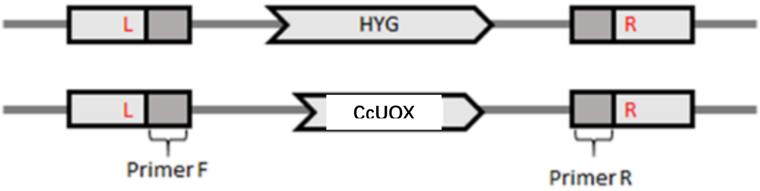

B

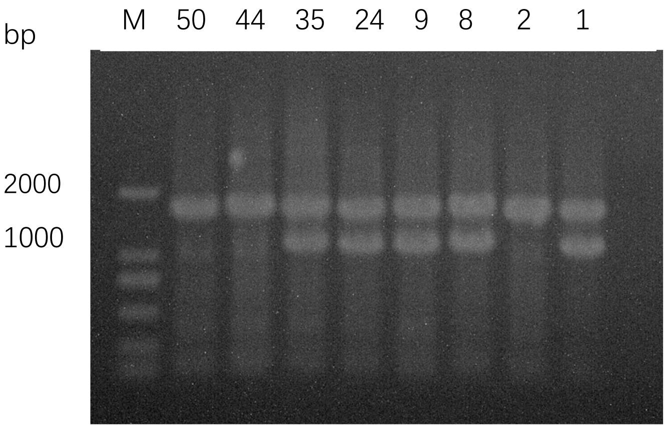

Figure S2. Construction and conformation of *CcUOX* mutants.

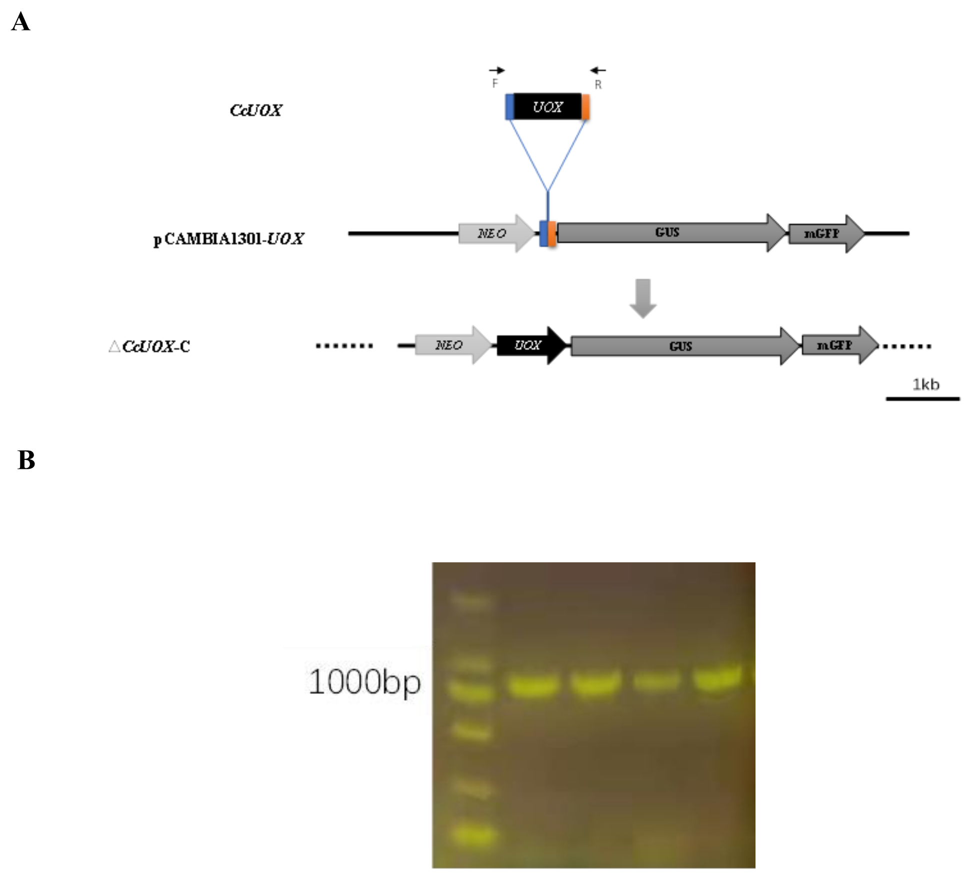

Figure S3. Construction and conformation of *CcUOX* complement lines.

Table S1. Primers used in this study.

Name	Forward primer (5' – 3')	Reverse primer (5' – 3')
DN13423_c0_g1	TCTTTGTTCTTGCCAGCCTG	AATCGTGCGAAAGCCACTT
DN9931_c0_g2	TCCCTCCTCCTGACTCTCCT	TGGATCAATGTTGTCACGGA
DN3418_c0_g3	TGCCAACGGACAGAATGC	TCAGCATCAGAATTGGGTTTC
DN6581_c0_g1	TAAATCCTCCATTCTCGCCC	TTGACGGTTATGACAGGAACG
DN12164_c0_g1	GCCATCTTCAGAGCTTTGGA	TTTGCTCATCCGTGTACGC
DN226_c0_g1	TCCTGGTGTGTTGGCTCAA	AGTCCAGTGACGAAGGCAAC
DN11890_c1_g15	AGTGCCAACAACTCCATTCAA	ACGGTAAATCTTGGAAGGGC
DN11849_c2_g29	GACTGTTTCTGCCCTGACCTT	AACCGACTGCCGTACCAGA
DN7328_c0_g2	TCCCTCCTCCTGACTCTCCT	TGTGACCTGCTGGATCAATGT
DN9931_c0_g2	TCCCTCCTCCTGACTCTCCT	TGGATCAATGTTGTCACGGA
DN11173_c0_g7	GCAAGGTTGGTCTTTACGCA	CATCTCGACGCAAGGGATT
DN9691_c0_g4	GCGATGGGAAATAATGGGAG	CGCTGCTTGCTGACTGTGT
DN14019_c0_g1	CGAACGGTCTCATCAGGTG	TCCAACCTCTCCGTCTCACTC
CcACT	GTTTCGCCGGTGACGATG	CTGGCCCATACCAATCATGA
Up-CcUOX	CGGAATTCTGAGATGAACCAACAGACC	CGGGGTACCGTTGGCGGTTGTGGTTG
Down-CcUOX	CGGGATCCGTTGGGGCGCTGTTGAAA	ACGCGTCGACAGAGGAAGAGAGCCGTGA
C-CcUOX	GAAGATCTATGCCCGTTCTCGCCTC	GAGGGGGGGCCCCAACTTGGCCGTCTCGT

Table S2. QC analysis of raw RNA-seq data.

Sample	Raw_Reads	Raw_Bases	Q20%	Q30%	GC%
CCK_1	54393274	8.21G	96.99	92.65	55.86
CCK_2	46981560	7.09G	96.74	92.36	56.52
CCK_3	57714152	8.71G	97.60	94.00	55.19
CT24_1	49376802	7.46G	96.72	91.95	56.46
CT24_2	56565410	8.48G	97.29	93.34	56.98
CT24_3	45635664	6.89G	96.64	91.81	56.73

Table S3. QC analysis of clean RNA-seq reads.

Sample	Raw_Reads	Raw_Bases	Valid_Reads	Valid_Bases	Valid%	Q20%	Q30%	GC%
CCK_1	54393274	8.21G	53240610	7.84G	97.88	98.13	94.69	55.82
CCK_2	46981560	7.09G	45645998	6.72G	97.16	98.30	94.99	56.44
CCK_3	57714152	8.71G	56811730	8.41G	98.44	98.45	95.54	55.16
CT24_1	49376802	7.46G	48285706	7.10G	97.79	97.93	94.10	56.43
CT24_2	56565410	8.48G	55774962	8.18G	98.60	98.47	95.28	56.95
CT24_3	45635664	6.89G	44600614	6.55G	97.73	97.89	94.04	56.69

Table S4. Differentially expressed genes encoding transporters in *C. camelliae* during infection tea plants.

Gene_ID	Annotation	log2FC	regulation
TRINITY_DN11973_c4_g8	ABC metal ion transporter	1.6823	up
TRINITY_DN11173_c0_g7	ABC multidrug transporter	2.0876	up
TRINITY_DN12055_c2_g1	ABC multidrug transporter	1.5796	up
TRINITY_DN12059_c0_g5	ABC multidrug transporter	-1.2498	down
TRINITY_DN12086_c2_g31	ABC multidrug transporter mdr1	-1.873	down
TRINITY_DN321_c0_g1	ABC transporter	5.1363	up
TRINITY_DN11894_c2_g1	ABC transporter	-1.7989	down
TRINITY_DN12195_c0_g1	ABC transporter	-3.254	down
TRINITY_DN321_c0_g2	ABC transporter	6.6215	up
TRINITY_DN13723_c0_g1	ABC transporter	1.2063	up
TRINITY_DN11890_c1_g15	ABC transporter	2.5839	up
TRINITY_DN8932_c0_g2	ABC transporter cdr4	-1.863	down
TRINITY_DN226_c0_g1	ABC-2 type transporter	3.3179	up
TRINITY_DN11824_c0_g10	ABC-2 type transporter	1.7347	up
TRINITY_DN8932_c0_g3	ABC-2 type transporter	-5.0486	down
TRINITY_DN4309_c0_g1	ABC-2 type transporter	-6.5303	down
TRINITY_DN11699_c1_g24	MFS transporter	6.6793	up
TRINITY_DN15097_c0_g1	MFS transporter	4.1204	up
TRINITY_DN3861_c0_g1	MFS transporter	3.5805	up
TRINITY_DN9466_c1_g2	MFS transporter	2.7175	up
TRINITY_DN11663_c0_g5	MFS transporter	2.5509	up
TRINITY_DN3451_c0_g1	MFS transporter	2.4926	up
TRINITY_DN11921_c3_g15	MFS transporter	2.481	up
TRINITY_DN12040_c2_g17	MFS transporter	2.4302	up
TRINITY_DN10590_c0_g4	MFS transporter	1.8585	up
TRINITY_DN6033_c0_g1	MFS transporter	1.7487	up
TRINITY_DN4368_c0_g1	MFS transporter	1.5483	up
TRINITY_DN13457_c0_g1	MFS transporter	1.2817	up
TRINITY_DN12370_c0_g1	MFS transporter	1.0819	up
TRINITY_DN11696_c2_g7	MFS transporter	1.032	up
TRINITY_DN11833_c0_g4	MFS transporter	-1.1364	down
TRINITY_DN1372_c0_g1	MFS transporter	-1.2803	down
TRINITY_DN12064_c3_g9	MFS transporter	-1.6753	down
TRINITY_DN12529_c0_g1	MFS transporter	-2.2225	down
TRINITY_DN6370_c0_g3	MFS transporter	-2.2813	up
TRINITY_DN11572_c0_g3	MFS transporter	-2.7393	down
TRINITY_DN366_c0_g1	MFS transporter	-2.9501	down
TRINITY_DN11716_c1_g12	MFS transporter	-2.9876	down
TRINITY_DN11999_c3_g28	MFS transporter	-3.8719	up
TRINITY_DN11211_c0_g3	MFS transporter	-4.1074	down

TRINITY_DN7649_c0_g2	MFS transporter	-4.1259	down
TRINITY_DN3814_c0_g1	MFS transporter	-4.5397	down
TRINITY_DN1345_c0_g1	MFS transporter	-4.694	down
TRINITY_DN7196_c0_g1	MFS transporter	6.0658	up
TRINITY_DN11697_c1_g2	MFS transporter	4.8649	up
TRINITY_DN6058_c0_g1	MFS transporter	4.3546	up
TRINITY_DN12297_c0_g1	MFS transporter	2.8969	up
TRINITY_DN713_c0_g1	MFS transporter	1.9625	up
TRINITY_DN8102_c0_g2	MFS transporter	1.6065	up
TRINITY_DN11873_c0_g3	MFS transporter	-1.5616	down
TRINITY_DN3687_c0_g3	MFS transporter	1.7476	up
TRINITY_DN14935_c0_g1	MFS alpha-glucoside transporter	3.28	up
TRINITY_DN11687_c0_g3	MFS hexose transporter	1.8525	up
TRINITY_DN15082_c0_g1	MFS monosaccharide transporter	1.4955	up
TRINITY_DN857_c0_g1	MFS multidrug transporter	8.6864	up
TRINITY_DN4788_c0_g1	MFS multidrug transporter	3.079	up
TRINITY_DN4033_c0_g1	MFS multidrug transporter	2.2586	up
TRINITY_DN12095_c0_g1	MFS multidrug transporter	1.671	up
TRINITY_DN13961_c0_g1	MFS multidrug transporter	-1.1098	down
TRINITY_DN11995_c0_g24	MFS multidrug transporter	-1.117	down
TRINITY_DN9590_c0_g2	MFS multidrug transporter	-5.782	down
TRINITY_DN2988_c0_g1	MFS phospholipid transporter git1	-4.44	down
TRINITY_DN2988_c0_g2	MFS phospholipid transporter git1	-4.9064	down
TRINITY_DN6904_c0_g1	MFS quinate transporter	3.7487	up
